# A bifurcation study to guide the design of a landing gear with a combined uplock/downlock mechanism

**DOI:** 10.1098/rspa.2014.0332

**Published:** 2014-12-08

**Authors:** James A. C. Knowles, Mark H. Lowenberg, Simon A. Neild, Bernd Krauskopf

**Affiliations:** 1 Department of Aeronautical and Automotive Engineering, Loughborough University, Loughborough LE11 3TU, UK; 2 Department of Aerospace Engineering, University of Bristol, Bristol BS8 1TR, UK; 3 Department of Mathematics, University of Auckland, Auckland 1142, New Zealand

**Keywords:** landing gear mechanism, numerical continuation, bifurcation analysis

## Abstract

This paper discusses the insights that a bifurcation analysis can provide when designing mechanisms. A model, in the form of a set of coupled steady-state equations, can be derived to describe the mechanism. Solutions to this model can be traced through the mechanism's state versus parameter space via numerical continuation, under the simultaneous variation of one or more parameters. With this approach, crucial features in the response surface, such as bifurcation points, can be identified. By numerically continuing these points in the appropriate parameter space, the resulting bifurcation diagram can be used to guide parameter selection and optimization. In this paper, we demonstrate the potential of this technique by considering an aircraft nose landing gear, with a novel locking strategy that uses a combined uplock/downlock mechanism. The landing gear is locked when in the retracted or deployed states. Transitions between these locked states and the unlocked state (where the landing gear is a mechanism) are shown to depend upon the positions of two fold point bifurcations. By performing a two-parameter continuation, the critical points are traced to identify operational boundaries. Following the variation of the fold points through parameter space, a minimum spring stiffness is identified that enables the landing gear to be locked in the retracted state. The bifurcation analysis also shows that the unlocking of a retracted landing gear should use an unlock force measure, rather than a position indicator, to de-couple the effects of the retraction and locking actuators. Overall, the study demonstrates that bifurcation analysis can enhance the understanding of the influence of design choices over a wide operating range where nonlinearity is significant.

## 1. Introduction

Mechanism design processes tend to focus on determining paths for key parts of the mechanism [1–3]. If the path is the only desired output, specific methods to compute the kinematics can be used [4,5]. These types of approaches are prevalent in robotics applications—an area in which much of the current mechanism research occurs [6,7]. When the dynamics of the mechanism along a path is required, a multibody dynamics-based formulation tends to be sought [8–13]. A multibody dynamics approach is often used in a wide variety of engineering applications, of which mechanism design forms a subset. Owing to the capabilities of multibody dynamics software packages to simulate dynamic systems in general (rather than just mechanisms), engineers working in an industrial context where mechanism analysis is not commonplace, will tend to use these methods during the design process.

Dynamical systems theory is a branch of mathematics that provides methods for the analysis of ordinary differential equations—see [14–16] for background information on this topic. The idea is that the underlying equilibria form the backbone of the system's dynamics. Knowledge of the underlying equilibria can therefore be used to understand various aspects of a given system's behaviour. One tool available to compute loci of equilibria is numerical continuation. This numerical tool enables points of interest to be traced, or ‘continued’, through the given model's state-parameter space. By considering how the system's equilibria (or other invariant objects) change under the variation of a parameter of interest, it is possible to build up a global picture of the structure of equilibria that govern the dynamic behaviour. Several examples in the literature have shown the benefits offered by this approach for the analysis of engineering systems: with applications in aerospace [17–21], civil [22] and automotive engineering [23,24], a dynamical systems approach has been shown to provide a useful, complementary tool, when paired with more traditional dynamic simulations.

The application of dynamical systems analysis methods in mechanism design and analysis is limited [25]. The field of kinematic mechanism analysis has seen the use of numerical continuation [26–28]; however, the key dynamical systems concept of considering how bifurcations populate the model parameter space appears to be distinct from all of these applications. Part of the reason why dynamical systems concepts have not been applied so readily to mechanism problems may be attributed to the natural mathematical form that the dynamics of constrained motion adopts. A mechanism's motion can be expressed naturally as a differential algebraic equation (DAE). These equations can be transformed into ODEs by appropriate differentiation of the constraints [29]. Numerical continuation can then be applied to the resulting system of equations most effectively when minimal coordinates are used. Without expressing the system in minimal coordinates, the continuation algorithm requires additional parameters to numerically drive relationships between coordinates to zero, in analogy with ‘unfolding parameters’ used to compute solutions of conservative systems [30]. Arguably, these difficulties have limited the use of numerical continuation to multibody dynamic analysis but have been addressed in recent publications, including [31,32].

This paper considers an industrially relevant application of numerical continuation methods to mechanism design. Specifically, the analysis of an aircraft nose landing gear (NLG) with a single uplock/downlock mechanism for securing the NLG in its retracted/deployed states is analysed. This type of NLG locking mechanism has not been analysed in the literature, but it is known to be highly sensitive to the application of different actuator forces when using a conventional position indicator to schedule the unlock and retraction actuators. An understanding of its behaviour is essential for guiding design decisions, in particular, for the development of an appropriate control schedule for the actuator forces acting throughout the extension/retraction cycle.

Conventionally, when deployed, a locking mechanism is engaged, via lock springs, to fix the landing gear in the deployed state: here, it is said to be *downlocked*. When the NLG is required to retract, this locking mechanism is released with an unlock actuator. A retraction actuator then moves the landing gear between the deployed and retracted states. At the end of the retraction cycle, the NLG needs to be fixed in the retracted state—at this point, it is said to be *uplocked*. This is usually achieved with the use of a dedicated uplock mechanism and associated (third) actuator, which clamps the landing gear in its retracted position [33–35]. In this paper, a novel alternative to this typical landing gear system is considered. The alternative landing gear system uses the same locking mechanism to uplock (as well as downlock) the NLG, through clever mechanism design. This results in the need for only two actuators: a retraction actuator and an unlock actuator. For this locking strategy, the springs used to engage the lock mechanism when the NLG is deployed (when its weight works with the spring force) must provide enough force to engage the uplock mechanism when the landing gear is retracted (and its weight opposes the spring force). It is therefore of critical design importance to know if the lock springs can provide sufficient force to uplock the NLG in the stowed position, while keeping the spring stiffness low to allow a small unlock actuator to be used.

A landing gear model consisting of a set of coupled kinematic and force equilibrium equations was presented in [31,32]. In these papers, the effect of the location of the lock springs and retraction actuator placement, respectively, for a landing gear with a traditional locking mechanism was analysed with the tool of continuation. This model is summarized in §2. A reduced version of the NLG model, created by just considering the kinematic equations from the full model, is used to portray the mechanism kinematics in §3—this approach is akin to standard kinematic applications of numerical continuation [26–28]. Here, the numerical continuation package AUTO [36] is used via the Dynamical Systems Toolbox in Matlab [37]. The bifurcation analysis, presented in §4, examines the landing gear extension and retraction process from a dynamical systems perspective. The landing gear is assumed to be attached to an aircraft in a steady state; dynamic loading on the gear is not considered here and remains an interesting topic for future research. Topological changes in the bifurcation diagrams as the unlocking force changes are identified. Analysis of these changes is used to specify the minimum stiffness of the lock springs that achieves both uplock and downlock. In addition, this provides insight into the reasons behind coupling effects of the unlock and retraction actuators when unlocking from the retracted state. Section 5 provides some concluding remarks.

## 2. Model of the nose landing gear mechanism

The equation formulation is based upon a previously published model of a planar NLG mechanism [31], but with some necessary adjustments specific to the present system. These adjustments are the inclusion of additional external forces, such as the retraction and unlock actuator forces, as well as a different geometry associated with the downlock spring placement. Because of this, the overall equations are sufficiently different to warrant their presentation in this work.

**Figure 1.**
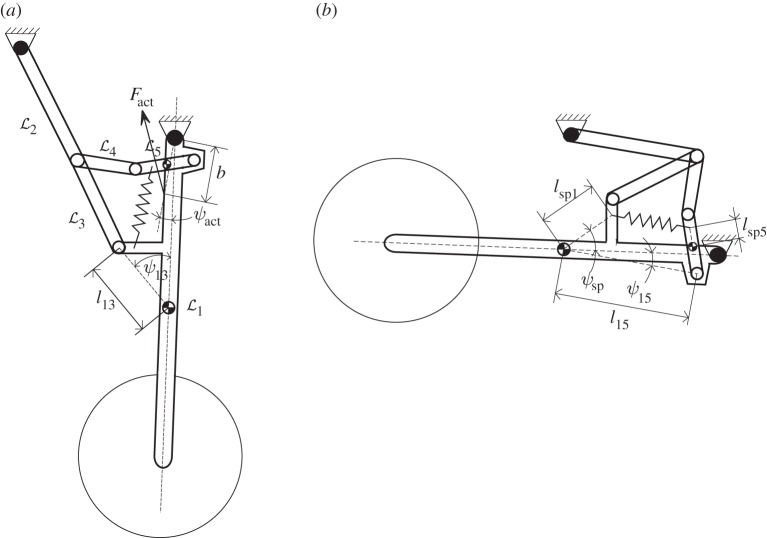
Schematic of the NLG mechanism: (*a*) deployed and (*b*) retracted.

Figure 1 shows the landing gear mechanism in both the deployed (*a*) and retracted (*b*) states. Each of the five links is assumed to be a rigid body, joined to one another by planar rotational joints (white circles). The shock strut and upper drag stay, links $L_{1}$ and $L_{2}$, respectively, are connected to two fixed attachment points on the aircraft, also via planar rotational joints (black circles).

The model is derived using Newtonian mechanics, assuming that each link must be in equilibrium (when considered as a free body) for the overall mechanism to be in equilibrium. There are 14 kinematic position equations and 19 force equilibrium equations, which are expressed in terms of 33 unknown states (corresponding to link positions, rotations and forces). Considering every link individually would yield 23 force equilibrium equations; however, as the forces at the attachment points are not of interest for this study, four equations (corresponding to horizontal and vertical resolution of links $L_{1}$ and $L_{2}$) can be removed. No further reductions are made beyond this: all inter-link forces are included in the continuation to avoid the need to post-process any results.

Since the derivation of the model has been presented before [31], only the final 33 equations are shown here. The 14 geometric constraints are

2.1 $$\begin{array}{rl} x_{2}-A_{x}-\frac{L_{2}}{2}\cos\theta_{2} & =0,\\ y_{2}-A_{y}-\frac{L_{2}}{2}\sin\theta_{2} & =0,\\ x_{2}-x_{3}+\frac{L_{2}}{2}\cos\theta_{2}+\frac{L_{3}}{2}\cos\theta_{3} & =0,\\ y_{2}-y_{3}+\frac{L_{2}}{2}\sin\theta_{2}+\frac{L_{3}}{2}\sin\theta_{3} & =0,\\ x_{3}-x_{1}+\frac{L_{3}}{2}\cos\theta_{3}-l_{13}\cos\left(\theta_{1}+\psi_{13}\right) & =0,\\ y_{3}-y_{1}+\frac{L_{3}}{2}\sin\theta_{3}-l_{13}\sin\left(\theta_{1}+\psi_{13}\right) & =0,\\ x_{1}+\frac{L_{1}}{2}\cos\theta_{1} & =0,\\ y_{1}+\frac{L_{1}}{2}\sin\theta_{1} & =0,\\ x_{2}-x_{4}+\frac{L_{2}}{2}\cos\theta_{2}+\frac{L_{4}}{2}\cos\theta_{4} & =0,\\ y_{2}-y_{4}+\frac{L_{2}}{2}\sin\theta_{2}+\frac{L_{4}}{2}\sin\theta_{4} & =0,\\ x_{4}-x_{5}+\frac{L_{4}}{2}\cos\theta_{4}+\frac{L_{5}}{2}\cos\theta_{5} & =0,\\ y_{4}-y_{5}+\frac{L_{4}}{2}\sin\theta_{4}+\frac{L_{5}}{2}\sin\theta_{5} & =0,\\ x_{5}-x_{1}+\frac{L_{5}}{2}\cos\theta_{5}-l_{15}\cos\left(\theta_{1}+\psi_{15}\right) & =0\\ \mathrm{and}\;y_{5}-y_{1}+\frac{L_{5}}{2}\sin\theta_{5}-l_{15}\sin\left(\theta_{1}+\psi_{15}\right) & =0, \end{array}\}$$

where link $L_{i}$, of length *L*_*i*_, is at an angle *θ*_*i*_ to the horizontal, with centre of gravity (cg) coordinates (*x*_*i*_,*y*_*i*_) that are positioned at the midpoint of all links. The upper drag stay (link $L_{2}$) and the shock strut (link $L_{1}$) are attached to the airframe at points (*A*_*x*_,*A*_*y*_) and (0,0), respectively. All other elements are indicated in figure 1.

The various elements within the force/moment equilibrium equations can be expressed in the matrix form

2.2 $$\text{A}\bar{F}-\bar{B}=0,$$

where $\bar{F}$ is a vector of the inter-link forces, **A** is a matrix of force coefficients and $\bar{B}$ is a vector of the remaining terms—namely the spring, actuator and gravitational forces. The ingredients of equation (2.2) are given in appendix A.

It should be noted that in vector $\bar{B}$ the influence of the retraction actuator is expressed in terms of forces $F_{\mathrm{ret}}^{x}$ and $F_{\mathrm{ret}}^{y}$. Previous work has shown that the underlying equilibria in a retraction cycle are influenced by the position of the retraction actuator [32]. In order to remove the influence of actuator position on the results, the moment (about the origin) applied by the retraction actuator on the main strut

2.3 $$M_{\mathrm{ret}}=-F_{\mathrm{ret}}^{y}b\cos(\theta_{1}+\psi_{\mathrm{act}})+F_{\mathrm{ret}}^{x}b\sin(\theta_{1}+\psi_{\mathrm{act}})$$

is used during the bifurcation analysis in §4. To allow for a direct, intuitive comparison with dynamic simulation, however, the results in §3 use the retraction force *F*_ret_ as the initial continuation parameter. The results in §3 are actuator position specific and are used primarily as a means to introduce the reader to the mechanism's motion from a bifurcation perspective.

## 3. Nose landing gear extension/retraction cycle

The use of a single locking mechanism to both downlock and uplock the NLG affects the range of motion that needs to be considered in any extension/retraction analysis. This increase in range of motion is particularly prevalent for the mechanism as modelled in this paper, because the model does not include the physical locklink stops that would be present in the real landing gear. Instead, the stops are incorporated as selected points in the results, but the system is allowed to move through them. It is, therefore, necessary to introduce the NLG mechanism motion first. The results presented in this section are designed to introduce the reader to the cyclical nature of an un-impeded NLG mechanism (i.e. one without locklink stops); the kinematics are considered first, before results including landing gear forces are presented.

### (a) Mechanism kinematics

**Figure 2.**
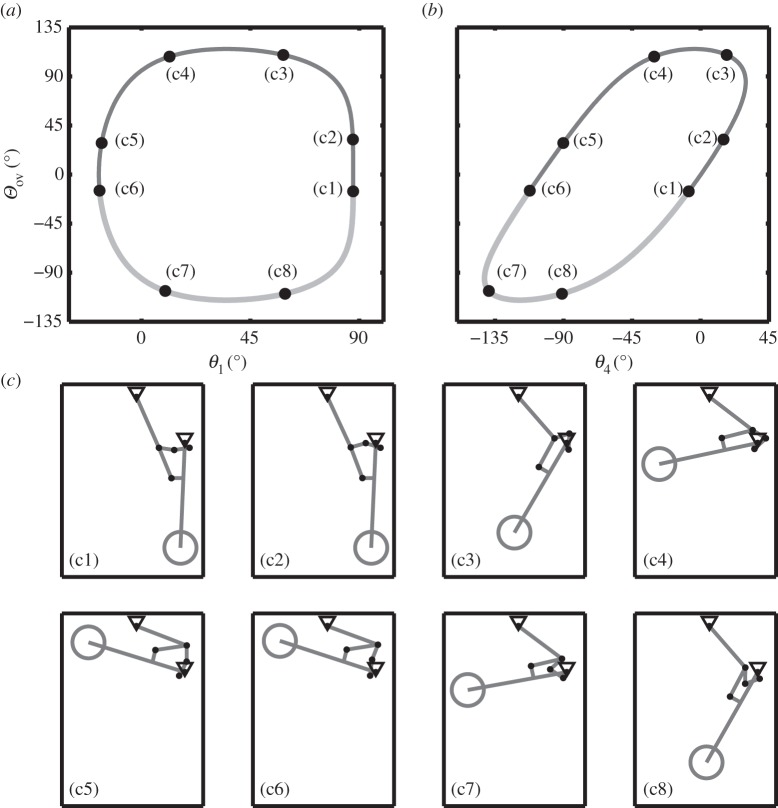
Kinematic solution for motions of the NLG. The projections show how overcentre angle *Θ*_ov_ varies as a function of (*a*) shock strut angle *θ*_1_ and (*b*) locklink angle *θ*_4_. The light grey part of the curve indicates solutions beyond the stops. Eight NLG positions are indicated in panels (c1)–(c8). Rotational joints are denoted by black dots, and the triangles indicate the landing gear attachment points. Panels (c1) and (c6) show the deployed and retracted landing gear, respectively.

By considering only the 14 geometric constraint equations of the model, equation (2.1), it is possible to solve an algebraic system that represents the kinematics of the NLG mechanism, i.e. the position relationships between all interlinked model coordinates. Equilibria of the resulting reduced-order system can be found and continued very efficiently, and their presentation allows the visualization of the NLG motion. Figure 2 presents the kinematic results, which describe the landing gear's cyclical motion when no locklink stops are present. The landing gear's one degree of freedom is represented as a single solution curve in the 15-dimensional state space of the kinematic model: figure 2*a*,*b* shows two projections of this solution curve, onto the (*Θ*_ov_,*θ*_1_)-plane and the (*Θ*_ov_,*θ*_4_)-plane, respectively. The overcentre angle *Θ*_ov_ represents the angle between the two locklinks; it is defined as *Θ*_ov_=*θ*_4_−*θ*_5_ and is (hence) zero when the two locklinks lie parallel to one another (as a result of the sign convention used). For the NLG considered in this work, *Θ*_ov_=−15° has been defined as the locking point (i.e. the point where the locklink stops would make contact with one another—see panels (c1) and (c6) for the associated NLG positions). The retraction angle *θ*_1_ is the angle that the shock strut makes with the horizontal plane. It provides a clear indication of the position of the landing gear during a retraction cycle: if *θ*_1_<0°, it is near the retracted position; if *θ*_1_>80°, it is near the deployed position. By contrast, the locklink angle *θ*_4_ (as presented in figure 2*b*) cannot be used on its own to determine the position of the NLG within an extension or retraction cycle; however, it details the locklink positions near the locking points (c1) and (c6).

The dark section of the curve in figure 2*a*,*b* shows solutions that are accessible to the NLG mechanism's motion even when locklink stops are included. By contrast, the light grey section of the curve indicates solutions that would be unobtainable in an NLG with locklink stops. Points (c1)–(c8) correspond to NLG positions shown in panels (c1)–(c8), respectively. It can be seen that points (c1) and (c6) lie on the interface between the dark and light curves—these therefore correspond to the deployed and retracted landing gear positions, respectively. While *Θ*_ov_ also provides an indication of the locklinks' position near the locking points, it is not possible to distinguish between the downlock and uplock points from this quantity alone. For this reason, the subsequent bifurcation analysis will consider the mechanism in terms of the locklink angle *θ*_4_.

It is possible to visualize the retraction process by careful consideration of the information presented in figure 2. The landing gear starts from the deployed, downlocked position, indicated by panel (c1). It can be seen that, from (c1) to (c2), only the locklinks appear to show much change in orientation within the mechanism. The solution curve in panel (*a*) supports this observation; it shows that the retraction angle remains virtually unchanged when moving from point (c1) to (c2), even though *Θ*_ov_ has changed quite considerably. This first step is the ‘unlocking’ phase, which would be performed by a small unlock actuator connected to one of the locklinks. The unlock actuator would continue to operate beyond (c2), but now the retraction actuator would also be engaged to help move the NLG from (c2) to (c3). The work from (c3) to (c4) would be performed entirely by the retraction actuator, and the unlock actuator would be ‘switched off’ in this part of the cycle. Once the NLG reaches (c5), the locking mechanism needs to be engaged once more, in order to uplock the landing gear. With sufficient force application from, for example, springs connected between locklink $L_{5}$ and the shock strut $L_{1}$ (figure 1), the locklinks can be engaged as the NLG moves from (c5) to (c6). Once at position (c6), the landing gear can be supported by the locklink stops and the retraction actuator can be ‘switched off’. Positions (c7) and (c8) in figure 2 would not be physically realizable in an actual NLG mechanism; they show how the mechanism moves beyond (c6) and back to (c1) if there are no locklink stops within the mechanism.

A similar process is applied to extend the landing gear from its retracted position (c6), where it follows the reverse of its extension back to point (c1). The unlock actuator is needed initially to unlock the landing gear (figure 2, (c6)–(c5)); however, it will not be able to unlock the mechanism on its own. As the whole weight of the NLG is now being supported by the locklinks, the retraction actuator needs to be engaged to support some of the weight of the NLG, such that the load on the locklinks is reduced. The correct application of this force is essential for the successful operation of the landing gear—it is investigated in more depth in §4*d*. Once unlocked, the landing gear extends from position (c5) to (c2), controlled by the retraction actuator. Locklink springs ensure that the mechanism reaches the deployed position at (c1) from (c2), where it is downlocked to enable the NLG to transfer ground loads into the airframe without collapsing.

As mentioned in the descriptions of the extension and retraction processes, the NLG requires some form of actuation to be able to move between its deployed and retracted states. To investigate the NLG mechanism behaviour under the action of actuation forces, it is necessary to consider next the full model as previously presented in §2.

### (b) Effect of actuator forces on nose landing gear equilibria

**Figure 3.**
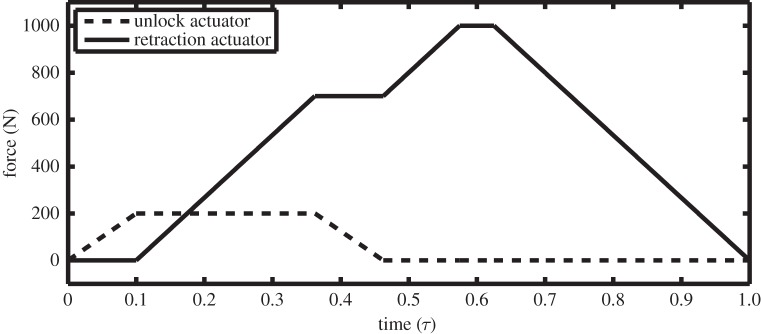
Actuator force schedules during a retraction cycle as a function of normalized time.

For a controlled extension or retraction to take place, the landing gear requires some form of actuation, applied in a swift, smooth manner. The majority of the retraction process is performed by the retraction actuator; however, at the start of the retraction cycle an unlock actuator is needed to unlock the mechanism. In a typical retraction cycle, the unlock actuator is engaged first, before being switched off part-way through the retraction cycle once the main retraction actuator is generating sufficient force to counteract the NLG load. For the dynamic simulations considered here, a simple actuator schedule was used that covers a retraction and uplocking; it is shown in figure 3.

The unlock actuator (dashed curve in figure 3) is engaged first, in order to unlock the NLG mechanism. Once unlocked, the retraction actuator (solid curve in figure 3) can be engaged. When the retraction actuator reaches a force value that is sufficient to support the landing gear in a given position, the unlock actuator force is reduced to 0. The reduction in unlock force would normally occur as the retraction force is increasing; however, for the demonstrative purpose in this work, the retraction force is held constant as the unlock force is reduced. With the unlock force at zero, the retraction actuator force is increased until the landing gear reaches the stowed position. Once it is stowed, the retraction actuator force is decreased to allow the locklinks to re-engage: this then fixes the NLG mechanism in place, and the actuator force can be reduced to zero.

The dynamic trajectory that the landing gear follows is heavily influenced by the structure of the underlying equilibria; as such, computing branches of these equilibria reveals useful insight into the dynamic response of the system. By considering the full model, equations (2.1) and (2.2), it is possible to determine relationships between externally applied forces (such as the retraction actuator force) and landing gear states (such as the locklink angle).

**Figure 4.**
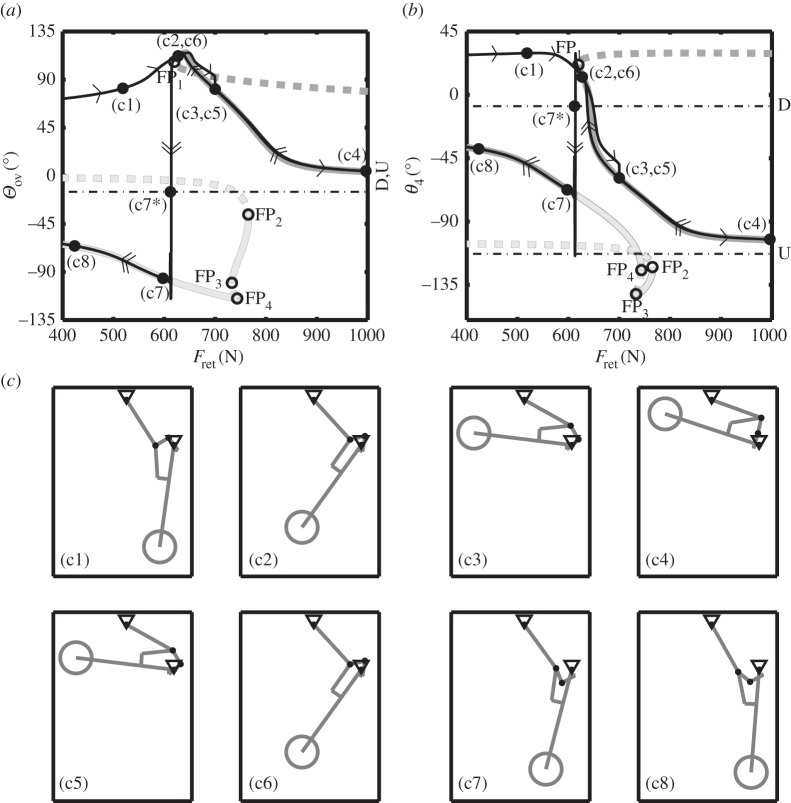
A comparison between numerical continuation results (thick, grey curves) and traditional, dynamic simulation (thin, black curve) with a downlock spring stiffness *k*=100 N m^−1^. Overcentre angle *Θ*_ov_ (*a*) and locklink angle *θ*_4_ (*b*) are shown as functions of retraction actuator force *F*_ret_. Single arrows on the dynamic simulation curve indicate the direction of motion for an increasing actuator force; double arrows indicate the motion for a decreasing actuator force. The continuation results were obtained for $F_{\mathrm{ul}}=0$ N. Solid curves represent dynamically stable equilibria, with dashed curves indicating unstable equilibria. The dark grey curve shows solutions where the locklinks are above overcentre, while the light grey curve indicates solutions where the locklinks are below overcentre. Fold points in the equilibrium curves (points FP_1_–FP_4_) are indicated by open circles. The thin, black dash-dotted curves D and U indicate the downlock and uplock angles, respectively. Panels (c1)–(c8) show the landing gear positions corresponding to the points indicated in panels (*a*) and (*b*).

Figure 4*a*,*b* shows the time history simulation trajectory (thin black curve) for the landing gear under the action of the actuator forces as depicted in figure 3. This trajectory is overlaid on a one-parameter bifurcation diagram with an unlock actuator force $F_{\mathrm{ul}}=0$, shown in two projections; figure 4*a* shows the projection in the (*Θ*_ov_, *F*_ret_)-plane, and figure 4*b* shows the projection in the (*θ*_4_, *F*_ret_)-plane. The thick dark grey curve corresponds to equilibria where the locklinks are above overcentre (i.e. *Θ*_ov_>0), and the thick light grey curve indicates equilibria where the locklinks are below overcentre (i.e. *Θ*_ov_<0). Unstable equilibria are indicated by dashed curves—the stability is inferred from dynamic simulation. As before, points (c1)–(c8) correspond to the NLG positions shown in panels (c1)–(c8). Open circles indicate fold point bifurcations, labelled FP_1_–FP_4_.

From the actuation schedule shown previously (figure 3), it can be seen that there is a period of time when both the unlock and retraction forces act. The unlock actuator force reached zero at point (c3) in figure 4—hence, the bifurcation diagram is only comparable for points (c3)–(c8), as it was computed for $F_{\mathrm{ul}}=0$.

Increasing the retraction actuator force slowly from point (c3) causes the dynamic trajectory to follow the curve of stable equilibria closely to (c4). The landing gear is maintained in this position by the retraction actuator force, before this force is slowly decreased. Decreasing the force causes the NLG to move back along the darkest curve of equilibria, from (c4) to (c6). It should be noted that positions (c3) and (c5) are the same; however, (c2) and (c6) just happen to appear at the same point in the two projections shown: while all the positional states are equivalent at both (c2) and (c6), there is a difference in unlock actuator force values between these two points.

Decreasing the force past (c6), the NLG follows the dark curve until the limit point bifurcation FP_1_. The equilibrium solution changes stability either side of this limit point, so rather than follow the unstable equilibria, the dynamic trajectory ‘jumps’ from the stable branch of the above-overcentre curve to the stable branch of the below-overcentre curve. As can be seen from considering panels (c6)–(c7) in figure 4, the transition from the dark grey, above-overcentre curve to the light grey, below-overcentre curve, indicates that the NLG has downlocked.

Some significant observations can be made in the light of the information presented in figure 4. The first is to note that, as *F*_ret_ was reduced from its value at (c4), the NLG mechanism was able to downlock successfully. This could be viewed in terms of a transition from the above- to below-overcentre curve, which was caused by a combination of gravitational and spring forces acting on the locklinks. At the other end of the retraction cycle, however, no such transition is possible with the chosen spring stiffness of 100 N m^−1^. The NLG mechanism therefore remains on the above-overcentre curve and is unable to uplock, i.e. reach the uplock position indicated by line U in figure 4. When approaching the retracted state, only the spring forces aid the locklinks in their quest to reach uplock; the gravitational forces oppose the locklinks' motion at position (c4), preventing the mechanism uplocking. Had the retraction force been increased beyond the range considered, the mechanism would not have moved from its position indicated in panel (c4). This is because mechanism configurations at overcentre (i.e. *Θ*_ov_=0) are singular in terms of the retraction force, meaning that an infinite amount of retraction actuator force is required to stow the landing gear.

One aspect of the NLG mechanism's motion that is not captured in the model, is the effect of the locklink stops. These stops limit the relative angular motion of the locklinks, such that overcentre angles of less than −15° cannot be reached. For an NLG mechanism with locklink stops, point (c7) in figure 4*a* would not have been reached. Instead, downlock would have occurred at point (c7*). This is because the locklink stops prevent the landing gear from reaching the stable branch on the below-overcentre curve by limiting the NLG's motion, creating a stable equilibrium solution along the downlocked line D. Once the NLG has reached point (c7*), it is in the position as shown in figure 2(c1), and will remain in this position irrespective of the amount of retraction actuator force applied, i.e. it is now downlocked. An unlock actuator is, hence, required to move the landing gear out of the deployed state; this process is discussed in §4*d*.

## 4. Bifurcation analysis of the extension/retraction cycle

The results shown in figure 4 demonstrate that the dynamic response is shaped by the underlying equilibria and, in particular, by the presence of fold bifurcations (labelled *FP*). These bifurcations dictate some key properties of the NLG mechanism's operation, including uplocking (in the retracted position) and unlocking (to allow the retraction or extension process to commence). This section analyses the transition from deployed to retracted states from a bifurcation perspective, with the emphasis placed on transitions to and from the retracted state (as this is the novel aspect of this NLG mechanism).

Previous work has shown that some fold bifurcations are dependent upon the positioning of the retraction actuator [32]—adjusting the actuator position relative to the NLG changes the effective moment arm of the actuator, which in turn affects the equilibrium solutions both quantitatively and qualitatively. As the focus of this paper is to investigate parameters directly associated with uplock and downlock, the moment exerted by the actuator to retract the landing gear (*M*_ret_, see equation (2.3)) is considered from now on, so that the results presented are independent of retraction actuator position. This means that, while qualitative ‘global’ differences will occur for particular actuator configurations when considering the whole retraction cycle, these differences will not affect the locking or unlocking behaviour.

### (a) Unlocking from deployment: the start of the retraction cycle

**Figure 5.**
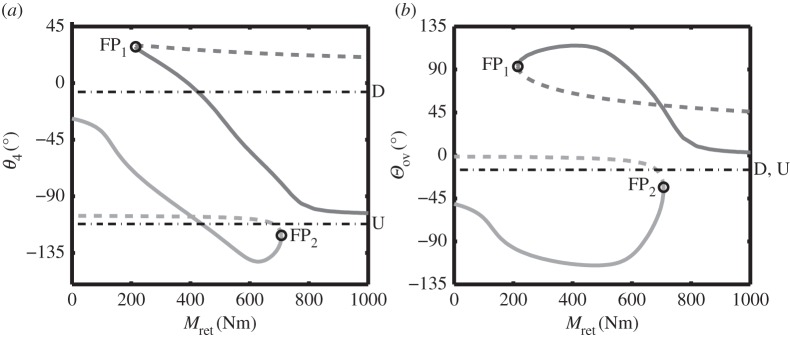
One-parameter bifurcation diagram in terms of retraction moment *M*_ret_ for the landing gear extension/retraction cycle, shown in terms of (*a*) locklink angle *θ*_4_ and (*b*) overcentre angle *Θ*_ov_. The results are for lock spring stiffness *k*=100 N m^−1^ and unlock actuator force $F_{\mathrm{ul}}=0$ N. Solid curves indicate stable solutions; dashed curves indicate unstable solutions. The dark grey curve shows solutions where the locklinks are above-overcentre, while the light grey curve indicates solutions where the locklinks are below-overcentre. Fold bifurcations are indicated by points FP_1_ and FP_2_. The black dotted-dashed lines, D and U, indicate the downlocked and uplocked positions (respectively) for the gear.

Figure 5 shows two projections of the equilibrium curves, computed for the baseline downlock spring stiffness of *k*=100 N m^−1^ and an unlock actuator force $F_{\mathrm{ul}}=0$ N. The projection in the (*M*_ret_,*θ*_4_)-plane is a different projection of the bifurcation diagram shown previously in figure 4, but now in terms of the retraction moment *M*_ret_. Note that in figure 5 two fold points remain, FP_1_ and FP_2_, which determine the landing gear's ability to move between a locked (either downlocked or uplocked) and an unlocked state.

The projection for figure 5*b* was chosen to facilitate relating the projection in figure 5*a* to the retracted and deployed positions, which occur at an overcentre angle *Θ*_ov_=−15°. By considering the two projections in figure 5 together, it is possible to identify and distinguish between the retracted and deployed points.

When deployed, the NLG is not acted upon by the retraction actuator. This means that *M*_ret_=0 at the start of the retraction cycle. It can be seen from Figure 5*a* that there is only one stable equilibrium solution at *M*_ret_=0, and that this equilibrium is part of the light grey, below-overcentre curve. Increasing the retraction moment causes the landing gear to follow the stable below-overcentre curve, until reaching the fold bifurcation at FP_2_, where it transitions from the below-overcentre to the above-overcentre curve. To reverse this transition, the retraction moment would need to be decreased past FP_1_, where the NLG would behave as shown previously in figure 4.

The presence of locklink stops, however, would prevent the mechanism from moving along the below-overcentre curve, as motions along this curve require overcentre angles below *Θ*_ov_=−15° (figure 5*b*). The motion under the action of a retraction actuator alone would therefore be quite different from the process described above: it would not be able to move! In order to retract the landing gear, it is therefore necessary to move onto the dark grey, above-overcentre curve. This is achieved through the use of the unlock actuator.

**Figure 6.**
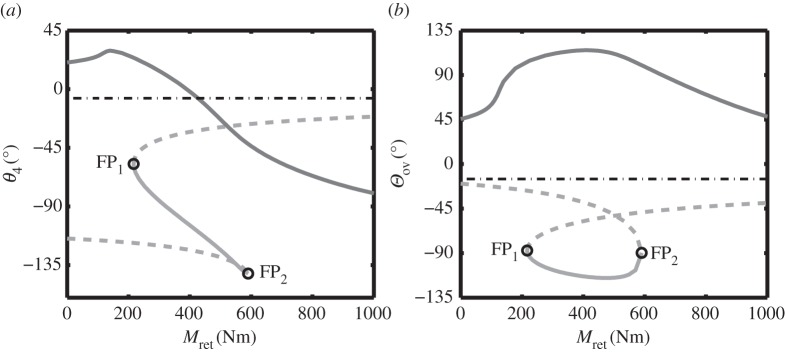
One-parameter bifurcation diagram in terms of retraction moment *M*_ret_ for the landing gear extension/retraction cycle, shown in terms of (*a*) locklink angle *θ*_4_ and (*b*) overcentre angle *Θ*_ov_. The results are for lock spring stiffness *k*=100 N m^−1^ and unlock actuator force $F_{\mathrm{ul}}=450$ N. Solid curves indicate stable solutions; dashed curves indicate unstable solutions. The dark grey curve shows solutions where the locklinks are above-overcentre, while the light grey curve indicates solutions where the locklinks are below-overcentre. Fold bifurcations are indicated by points FP_1_ and FP_2_. The black dotted-dashed line indicates the downlocked position for the gear.

Figure 6 shows how the equilibria depicted in figure 5 change with the application of an unlock actuator force of $F_{\mathrm{ul}}=450$ N. Now, the single stable equilibrium point at *M*_ret_=0 is part of the above-overcentre curve. This means that increasing the retraction moment causes the NLG to follow the dark grey equilibria curve, so the NLG can be retracted.

**Figure 7.**
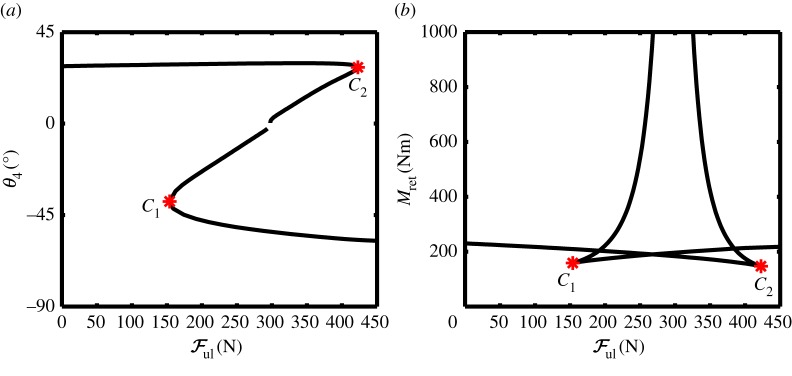
Two-parameter bifurcation diagrams of the downlock limit point FP_1_, showing (*a*) locklink angle *θ*_4_ and (*b*) retraction moment *M*_ret_ as functions of unlock actuator force $F_{\mathrm{ul}}$. Points *C*_1_ and *C*_2_ are cusp bifurcations. (Online version in colour.)

The ability to be able to retract the landing gear is linked to the transition of the fold bifurcation FP_1_, from the above- to below-overcentre curve. Figure 7 shows two-parameter continuations of the fold point FP_1_ in the ($F_{\mathrm{ul}},\theta_{4}$)-plane and the ($F_{\mathrm{ul}},M_{\mathrm{ret}}$)-plane. There are actually two separate curves of fold point FP_1_ with a cusp point, *C*_1_ and *C*_2_, on each fold curve. The gap dividing the fold locus into two parts in figure 7*a* is due to the fact that the retraction moment *M*_ret_ increased towards infinity as the unlock force approached an asymptotic limit (corresponding to the locklinks becoming aligned—a geometric singularity in the mechanism). Because of this, the two-parameter continuation of fold point FP_1_ was carried out in two parts; both up to values of *M*_ret_ beyond those shown in figure 7*b*.

For unlock force values $F_{\mathrm{ul}}<150$ N, the structure of equilibria is qualitatively similar to that in figure 5, whereas for $F_{\mathrm{ul}}>425$ N, the structure of equilibria is qualitatively similar to that in figure 6. As the unlock actuator force is increased from 150 to 425 N, the system undergoes a saddle transition—this transition will be discussed in more detail when considering landing gear uplocking, as it is this case that is the main focus of the paper.

In a real retraction process, the unlock actuator force reduces to zero as the main actuator retracts the NLG. This unlock actuator force reduction causes the branches of equilibria to change from the form shown in figure 6 to the form depicted in figure 5 (where $F_{\mathrm{ul}}=0$ N). When designing the actuation system, it is necessary to ensure that the unlock actuator is only switched off after the retraction actuator has reached a sufficiently high value such that the landing gear can be held in equilibrium on the above-overcentre curve in figure 5—approximately *M*_ret_=220 Nm for this NLG configuration.

Once on the above-overcentre curve in figure 5, however, the landing gear will stay on the above-overcentre curve if *M*_ret_ is continually increased. It is therefore not possible to achieve locking in the retracted state, as there is insufficient force provided by the lock springs. To achieve uplock, it is necessary to increase the spring force—the effect this has on the underlying equilibria will now be presented and discussed.

### (b) Effect of lock spring stiffness on nose landing gear uplocking

The lock springs in a landing gear are used to engage the locklinks, by providing a force to pull them between above- and below-overcentre. Gravity usually aides this process when the NLG downlocks; however, these springs have to work against gravity if the same mechanism is used to lock the landing gear in the retracted state. The critical case for sizing the lock springs is, therefore, the uplocking process.

**Figure 8.**
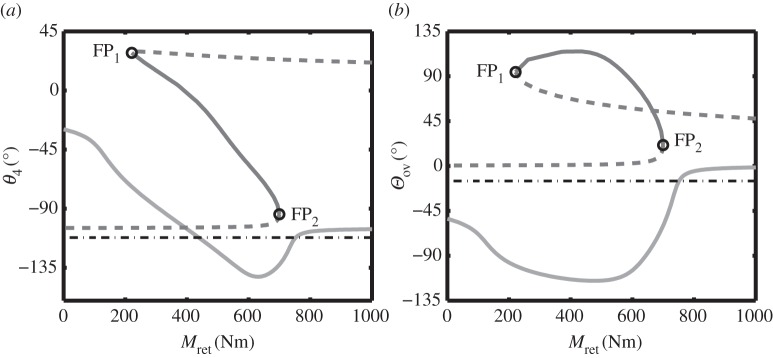
One-parameter bifurcation diagram in terms of retraction moment *M*_ret_ for the landing gear extension/retraction cycle, shown in terms of (*a*) locklink angle *θ*_4_ and (*b*) overcentre angle *Θ*_ov_. The results are for lock spring stiffness *k*=200 N m^−1^ and unlock actuator force $F_{\mathrm{ul}}=0$ N. Solid curves indicate stable solutions; dashed curves indicate unstable solutions. The dark grey curve shows solutions where the locklinks are above-overcentre, while the light grey curve indicates solutions where the locklinks are below-overcentre. Fold bifurcations are indicated by points FP_1_ and FP_2_. The black dotted-dashed line indicates the uplocked position for the gear.

Figure 8 shows a bifurcation diagram of the NLG extension/retraction cycle, where the lock springs have sufficient stiffness to stow the mechanism. Compared to figure 5, the lower fold point FP_2_ has been moved from the below-overcentre curve by increasing the spring stiffness. From a bifurcation perspective, this means that solutions that start on the below-overcentre curve will not be able to transition to the above-overcentre curve under the sole action of the retraction moment. By comparison, solutions from the stable branch of the above-overcentre curve will be able to reach the below-overcentre curve in two ways—by increasing *M*_ret_ above FP_2_, and by decreasing *M*_ret_ below FP_1_.

The implication of the bifurcation diagram in figure 8 for a real NLG mechanism is that the NLG with the higher stiffness lock springs can be uplocked: as the retraction moment increases, the landing gear (assuming it has been unlocked such that its initial condition is somewhere on the above-overcentre curve) will follow the dark grey above-overcentre curve until reaching FP_2_, when it will ‘jump’ down to the below-overcentre curve. Locklink stops would limit the motion of the landing gear along the below-overcentre curve, meaning that decreasing the retraction moment once below-overcentre would cause the NLG to uplock.

As the previous discussion regarding landing gear unlocking highlighted, uplocking appears to depend upon a fold point transitioning from one curve to the other—in this case, from below- to above-overcentre. Understanding this transition is crucial for determining the minimum required spring stiffness to achieve uplock. The process by which the fold point transfers from one curve to the other is examined next.

### (c) Two-parameter continuation of uplock fold point FP_2_

**Figure 9.**
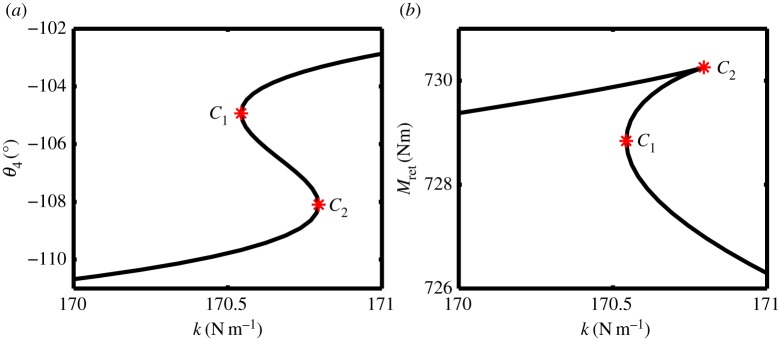
Two-parameter continuation of the uplock limit point FP_2_, showing (*a*) locklink angle *θ*_4_ and (*b*) retraction moment *M*_ret_ as functions of spring stiffness *k*. Points *C*_1_ and *C*_2_ are cusp bifurcations. (Online version in colour.)

Figure 9 shows two projections of the curve of uplock fold point FP_2_, in terms of (*a*), the landing gear state *θ*_4_ and spring stiffness *k*; (*b*), the two parameters used in the continuation, *M*_ret_ and *k*. Considering figure 9*a*, solutions for *k*<170.5 N m^−1^ correspond to retraction equilibria that are topologically equivalent to those shown in figure 5; the part of the fold curve where *k*>170.8 N m^−1^ corresponds to a landing gear configuration that can uplock, i.e. the retraction equilibria are topologically equivalent to figure 8. The transition between these two regions occurs through the mechanism of a transcritical bifurcation (or saddle transition), within a very small parameter range.

**Figure 10.**
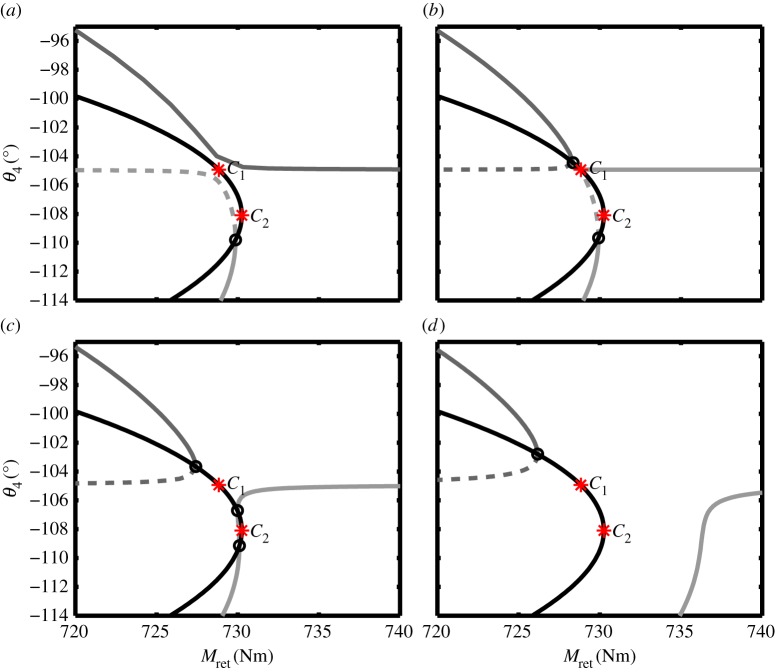
Bifurcation diagrams near the critical locklink spring stiffness, showing the transition in the curves of equilibria for spring stiffness of (*a*) *k*=170.496 N m^−1^, (*b*) *k*=170.543 N m^−1^, (*c*) *k*=170.698 N m^−1^ and (*d*) *k*=171.039 N m^−1^. In all four cases, $F_{\mathrm{ul}}=0$. As before, the dark grey curve corresponds to above-overcentre solutions, and the light grey curve indicates below-overcentre solutions. The black curve shows the locus of fold points from the two-parameter continuation of FP_2_ shown in figure 9. Fold points of the equilibria are indicated by black circles, with stars showing the codimension two cusp points in the fold curve (*C*_1_ and *C*_2_). (Online version in colour.)

Figure 10 shows enlarged bifurcation diagrams for four different spring stiffnesses around the uplock transition point, with the two-parameter continuation of the fold point FP_2_ (the black curve) plotted in each case. The case shown in panel (*a*) is qualitatively as that in figure 5—the curves of equilibria only intersect the locus of FP_2_ once, and this intersection occurs on the below-overcentre (light grey) curve.

Increasing the spring stiffness to just beyond the stiffness value at point *C*_1_, however, causes a qualitative change in the response; the equilibria are now as shown in figure 10*b*. The curves of equilibria now intersect the fold locus in three places—one on the above-overcentre curve, and the other two on the below-overcentre curve. The two additional fold points are created when the spring stiffness value passes the cusp point *C*_1_ in figure 9. At the cusp point, the system shows a transcritical bifurcation in the (*θ*_4_,*M*_ret_)-plane, as both the below- and above-overcentre curves become joined momentarily. Note that these curves connect differently before and after this bifurcation, which corresponds to the transition through a saddle point of the equilibrium surface in (*M*_ret_,*θ*_4_,*k*)-space [38].

Figure 10*c* produces a response that is qualitatively as the case in panel (*b*): while the two fold points that emerged in figure 10*b* have moved apart, the number of fold points in the parameter range considered remains unchanged. It is evident from panel (*c*) that the two fold points on the below-overcentre curve are moving together as the spring stiffness is increased. These two fold points coalesce when the spring stiffness reaches a critical value at point *C*_2_. As the spring stiffness undergoes a further slight increase beyond *C*_2_, there is a second qualitative change in the bifurcation diagram. Figure 10*d* shows that the response when the spring stiffness is increased just beyond its value at *C*_2_ is qualitatively similar to the response in figure 8 (where the landing gear is able to achieve uplock). As the response in panel (*d*) is qualitatively equivalent to a set of solutions where the landing gear can achieve uplock, the spring stiffness value at or above point *C*_2_ (figure 9) will result in a successful uplock.

It can also be reasoned that the landing gear would be able to lock in the retracted position for spring stiffnesses between *C*_1_ and *C*_2_. For the cases shown in figure 10*b*,*c*, the below-overcentre curve extends to the right-hand edge of the figure panel. As uplock can be viewed as a transition between the above-overcentre curve to the below-overcentre curve, it can be seen that the cases in both panels (*b*) and (*c*) correspond to a successful uplock. Because the case in panel (*a*) cannot achieve uplock, it can be seen that the spring stiffness at point *C*_1_ provides the absolute minimum stiffness required to achieve a successful uplock.

### (d) Effect of unlock actuator force on extension/retraction equilibria

When stowed, the locking actuator must be able to unlock the landing gear in order for it to deploy successfully. Unlike unlocking from the deployed state, the landing gear weight is supported by the locklinks in the retracted state. This means that an unlock actuator force, applied on its own, would need to overcome the whole weight of the landing gear in order to unlock from the retracted position. While this could theoretically be achieved with a very powerful unlock actuator, a more sensible solution is to use the retraction actuator to take most of the weight of the landing gear first. It is therefore necessary to consider any interactions that may occur between the unlock and retraction actuator forces, in order to control the extension process appropriately.

**Figure 11.**
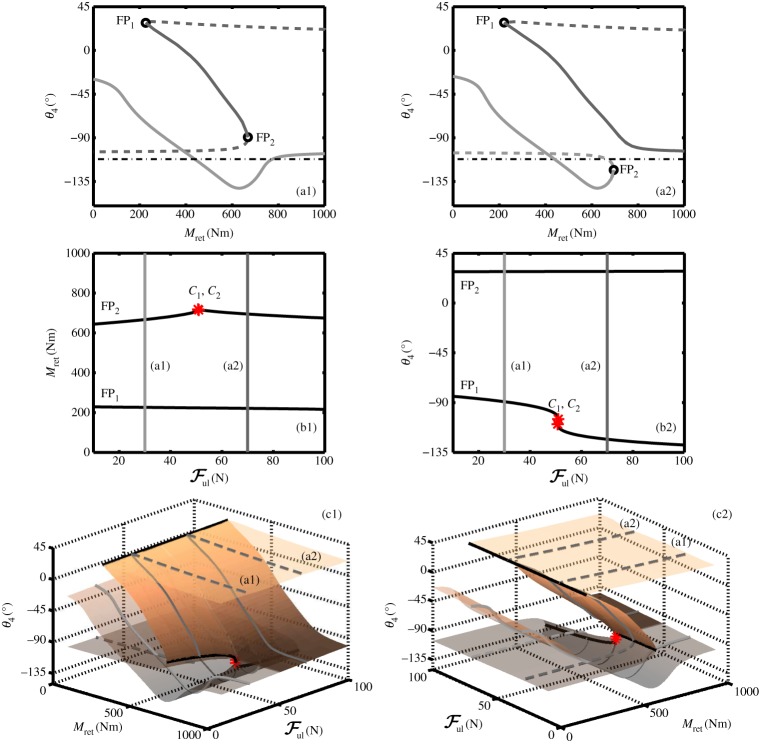
Effect of unlock actuator force $F_{\mathrm{ul}}$ on landing gear retraction for a constant downlock spring stiffness *k*=200 N m^−1^. Two unlock force cases are presented as one-parameter bifurcation diagrams: an unlock force $F_{\mathrm{ul}}=30$ N is used in (a1), and $F_{\mathrm{ul}}=70$ N in (a2). As before, fold bifurcations are indicated by black circles, and the stability of the solutions is indicated using solid curves for stable and dashed curves for unstable. Panels (b1) and (b2) show the fold curves FP_1_ and FP_2_ in parameter space, with the sections in panels (a1) and (a2) indicated by the grey lines. Cusp points *C*_1_ and *C*_2_ are indicated by stars. The surface of equilibria, shown from two viewpoints in (c1) and (c2), is in terms of retraction moment *M*_ret_, unlock actuator force $F_{\mathrm{ul}}$ and locklink angle *θ*_4_. The opaque section of the surface indicates stable, unlocked solutions. Fold bifurcations of the equilibria are indicated by the black curves, with two cusp points indicated by stars. The sections in panels (a1) and (a2) are indicated by grey curves. (Online version in colour.)

Figure 11(a1) and (a2) shows two qualitatively different bifurcation diagrams that occur for different values of the unlock actuator force $F_{\mathrm{ul}}$. They are comparable to figures 8*a* and 5*a* (respectively); however, the change in net locklink force between the two qualitative cases is now due to changing the unlock actuator force (rather than changing the spring stiffness). Figure 11(b1) shows the two loci of fold points in the ($F_{\mathrm{ul}},M_{\mathrm{ret}}$)-parameter plane, with two horizontal lines indicating the slices corresponding to panels (a1) and (a2). The transition mechanism between these qualitatively different slices is the reverse of the bifurcation mechanism by which locking in the retracted position is achieved—increasing unlock actuator force would cause a transition in the equilibria equivalent to moving from figure 10*d* to *a*. It is therefore reasoned that the transitions around the uplock point are a function of the net force acting on the locklinks, and that identifying the cusp bifurcation *C*_1_ would provide an absolute minimum unlock force value.

Figure 11 also provides a graphical representation of the unlocking process for a retracted landing gear. Panels (c1) and (c2) show a surface of retraction equilibria in terms of the retraction moment *M*_ret_, unlock force $F_{\mathrm{ul}}$ and locklink angle *θ*_4_. The locus of fold points FP_1_ and FP_2_ are indicated on the surface by the black curves; the two example parameter slices from panels (a1) and (a2) are indicated by the grey curves (a1) and (a2), respectively. Two different viewing angles for the surface are used to show how the above- and below-overcentre curves intersect one another.

Careful consideration of the surface in figure 11(c1) can provide some useful insights when defining an unlocking strategy for the NLG. Graphically, the process of unlocking a retracted landing gear can be viewed as moving the landing gear to a point on the opaque section of the surface. This is achieved through an appropriate, simultaneous application of the retraction and unlock actuator forces. Once the landing gear is on the opaque section of the curve, both actuator forces need to be reduced for it to deploy. A control strategy is therefore needed to schedule the actuator forces.

The simplest control strategy is to use a landing gear position indicator. A position indicator is conventionally used to define when the mechanism reaches the retracted state, so no additional measurements would be needed. The unlocked state of the NLG mechanism can be defined as a particular locklink angle, which once reached would cue a reduction in retraction actuator force to begin extending the landing gear.

The results in figure 11 highlight a complication with using the landing gear position to define when it is unlocked—specifying a geometric position for the NLG results in a coupling of the unlock and retraction actuator forces. Considering the case for a landing gear that specifies a value of *θ*_4_ as the locked/unlocked boundary (e.g. *θ*_4_=−95°), the control strategy effectively adds an extra boundary to the ‘unlocked’ region of the retraction surface. This new boundary is given by the intersection between the retraction surface and an imaginary horizontal plane, representing the ‘unlocked’ angle. Sections of the retraction surface below this unlocked angle correspond to uplocked solutions, while parts of the surface above the unlocked angle and on the opaque section of the surface correspond to unlocked solutions. The intersection between the unlocked angle and the retraction surface will determine the point in the NLG mechanism control system where the retraction moment can start to decrease.

The reason why this coupling occurs lies in the geometry of the NLG mechanism near overcentre. Motions working to retract the main shock strut (i.e. the motion induced by the retraction actuator) cause the locklinks to align, which is undesirable if the locklinks are slightly above overcentre (as increasing the retraction moment causes *θ*_4_ to decrease). Considering the lower edge of the surface in figure 11(c1) when *M*_ret_=1000 N, the value of *θ*_4_ for $F_{\mathrm{ul}}=0$ N is lower than the value of *θ*_4_ for $F_{\mathrm{ul}}=100$ N. The net effect is that, once the landing gear reaches the opaque section of the surface, increasing *M*_ret_ decreases *θ*_4_, while increasing $F_{\mathrm{ul}}$ increases *θ*_4_.

Knowledge of the retraction surface, figure 11*c*, suggests a different strategy for controlling the extension process. In a similar manner to the identification of a minimum spring stiffness, a minimum unlock actuator force can be identified by considering the lower locus of fold bifurcations. The equivalent point to point *C*_1_ on the fold curve of figure 9 provides the minimum unlock actuator force required to unlock the NLG from uplock. When the unlock actuator applies a force to the locklinks that is greater than the force indicated by point *C*_1_, the landing gear will be able to unlock from the retracted position. The retraction moment will need to be increased to beyond the value of the fold point for the given value of $F_{\mathrm{ul}}$, but once past this point the NLG will unlock as the retraction moment is decreased. Using the unlock actuator force (rather than the locklink position) to define when the landing gear is unlocked, de-couples the two actuator forces.

## 5. Concluding remarks

A bifurcation analysis approach has been used to inform mechanism design decisions, with application to a single uplock/downlock NLG mechanism. Numerical continuation applied to the constraint equations was shown to provide an efficient means of finding equilibria, and how they depend on different parameters of interest. Initial kinematic results were presented to aid visualization of the retraction/extension process, and included regions of motion normally inaccessible due to the presence of locklink stops.

Using the underlying idea that the equilibrium structure forms the backbone to specific dynamic behaviour of interest, the kinematic analysis was extended to include the effects of forces on and within the mechanism. A bifurcation diagram presented the mechanism response as a function of retraction actuator force (or moment). This was compared with an equivalent time history simulation, which showed good agreement between the two different methods, thus supporting the observation that the equilibria provide a backbone for the mechanism's dynamic behaviour. The time history simulation also allowed stability information to be inferred in the bifurcation diagram.

An in-depth bifurcation analysis was then conducted to determine how the equilibria change as functions of two important design parameters: the lock spring stiffness, which needs to be sufficient to engage the locklinks without being unnecessarily powerful (and hence heavy); and the unlock forces, which need to be scheduled appropriately to ensure successful operation, particularly when unlocking from the retracted position. Two qualitative changes in the bifurcation diagram were identified that allowed the landing gear to transition between the deployed and retracted states: an initial movement of fold point FP_1_ from the above- to below-overcentre curve (transition from downlocked to unlocked state) began the retraction cycle; the subsequent movement of fold point FP_2_ from the below- to above-overcentre curve (transition from unlocked to uplocked state) ensured the NLG could be uplocked. This second transition was identified as the crucial transition for successful operation of this novel mechanism.

By performing a two-parameter continuation of the fold point that affects uplocking, a minimum spring stiffness was identified that allows the landing gear to uplock. The process by which the equilibria change as a function of spring stiffness was analysed and shown to be a combination of two cusp bifurcations. Applying the unlock actuator force to unlock the landing gear from a stowed position reverses the transition of the loci of equilibria observed under spring stiffness variation. By considering the retraction surface in terms of retraction and unlock actuator forces, it was shown that the extension control methodology should use a force measure, rather than a position measure, to schedule the unlock forces.

Overall, the study presented here demonstrates that bifurcation analysis is a powerful tool for the investigation of mechanisms, such as the one considered here. In particular, the use of numerical continuation for the detection and continuation of bifurcations (fold points and cusp points in this work) and their representation in different parameter projections is useful for identifying boundaries for successful operation of a given mechanism, such as the NLG considered here. We argue that this kind of insight, which can be obtained quite quickly and at low cost, will be helpful for informing design decision, as well as for identifying regions of interest that can then be explored with dedicated simulations of high-fidelity but more complex models.

## Appendix A. Nose landing gear force/moment equilibrium equations

The various elements within the force/moment equilibrium equations can be expressed in the matrix form $\text{A}\bar{F}-\bar{B}=0$ where

A 1 $$\bar{B}=\left[\begin{array}{c} \frac{m_{2}}{2}g\cos\theta_{2}\\ 0\\ m_{3}g\\ \frac{m_{3}}{2}g\cos\theta_{3}\\ 0\\ m_{4}g\\ -\frac{m_{4}}{2}g\cos\theta_{4}\\ F_{sp5}^{x}\\ F_{sp5}^{y}+m_{5}g\\ \frac{m_{5}}{2}g\cos\theta_{5}+\frac{1}{2}F_{sp5}^{y}\left(1-\frac{l_{sp5}}{L_{5}}\right)\cos\theta_{5}-\frac{1}{2}F_{sp5}^{x}\left(1-\frac{l_{sp5}}{L_{5}}\right)\sin\theta_{5}\\ 0\\ 0\\ 0\\ 0\\ 0\\ 0\\ 0\\ 0\\ -\left(\frac{m_{1}}{2}g+m_{w}g\right)L_{1}\cos\theta_{1}+F_{sp1}^{x}\left[\frac{L_{1}}{2}\sin\theta_{1}-l_{sp1}\sin(\theta_{1}+\psi_{sp})\right]\\ -F_{sp1}^{y}\left[\frac{L_{1}}{2}\cos\theta_{1}-l_{sp1}\cos(\theta_{1}+\psi_{sp})\right]-F_{\mathrm{ret}}^{y}(b\cos(\theta_{1}+\psi_{\mathrm{act}}))\\ \;+\;F_{\mathrm{ret}}^{x}(b\sin(\theta_{1}+\psi_{\mathrm{act}}))+D \end{array}\right].$$

Here, *F*_sp_ is the spring force, with the subscript number indicating the link it acts on; *D* is the drag force, set to zero for this work; *F*_ret_ is the retraction actuator force; and all other quantities are as described before or as indicated in figure 1.

A 2 $${\bar{F}}^{T}=\begin{array}{ccccccccccc} {}[F_{2;3,4}^{x} & F_{2;3,4}^{y} & F_{3;2,4}^{x} & F_{3;2,4}^{y} & F_{3;1}^{x} & F_{3;1}^{y} & F_{1;3}^{x} & F_{1;3}^{y} & F_{1;5}^{x}\ldots & \\ F_{1;5}^{y} & F_{\mathrm{ul}} & F_{5;1}^{x} & F_{5;1}^{y} & F_{5;4}^{x} & F_{5;4}^{y} & F_{4;5}^{x} & F_{4;5}^{y} & F_{4;2,3}^{x} & F_{4;2,3}^{y}] & \end{array},$$

where the force exerted on the *i*th link by the *j*th link is given by *F*_*i*,*j*_, and $F_{\mathrm{ul}}$ is the magnitude of the unlock actuator force. Moreover,

A 3 $$\text{A}=\left[\begin{array}{ccccccccccccccccccc} -\sin\theta_{2} & \cos\theta_{2} & 0 & 0 & 0 & 0 & 0 & 0 & 0 & 0 & 0 & 0 & 0 & 0 & 0 & 0 & 0 & 0 & 0\\ 0 & 0 & 1 & 0 & 1 & 0 & 0 & 0 & 0 & 0 & 0 & 0 & 0 & 0 & 0 & 0 & 0 & 0 & 0\\ 0 & 0 & 0 & 1 & 0 & 1 & 0 & 0 & 0 & 0 & 0 & 0 & 0 & 0 & 0 & 0 & 0 & 0 & 0\\ 0 & 0 & 0 & 0 & -\sin\theta_{3} & \cos\theta_{3} & 0 & 0 & 0 & 0 & 0 & 0 & 0 & 0 & 0 & 0 & 0 & 0 & 0\\ 0 & 0 & 0 & 0 & 0 & 0 & 0 & 0 & 0 & 0 & 0 & 0 & 0 & 0 & 0 & 1 & 0 & 1 & 0\\ 0 & 0 & 0 & 0 & 0 & 0 & 0 & 0 & 0 & 0 & 0 & 0 & 0 & 0 & 0 & 0 & 1 & 0 & 1\\ 0 & 0 & 0 & 0 & 0 & 0 & 0 & 0 & 0 & 0 & 0 & 0 & 0 & 0 & 0 & -\sin\theta_{4} & \cos\theta_{4} & 0 & 0\\ 0 & 0 & 0 & 0 & 0 & 0 & 0 & 0 & 0 & 0 & F_{\mathrm{ul}5}^{x} & 1 & 0 & 1 & 0 & 0 & 0 & 0 & 0\\ 0 & 0 & 0 & 0 & 0 & 0 & 0 & 0 & 0 & 0 & F_{\mathrm{ul}5}^{y} & 0 & 1 & 0 & 1 & 0 & 0 & 0 & 0\\ 0 & 0 & 0 & 0 & 0 & 0 & 0 & 0 & 0 & 0 & \frac{l_{\mathrm{ul}5}}{L_{5}} & -\sin\theta_{5} & \cos\theta_{5} & 0 & 0 & 0 & 0 & 0 & 0\\ 1 & 0 & 1 & 0 & 0 & 0 & 0 & 0 & 0 & 0 & 0 & 0 & 0 & 0 & 0 & 0 & 0 & 1 & 0\\ 0 & 1 & 0 & 1 & 0 & 0 & 0 & 0 & 0 & 0 & 0 & 0 & 0 & 0 & 0 & 0 & 0 & 0 & 1\\ 0 & 0 & 0 & 0 & 1 & 0 & 1 & 0 & 0 & 0 & 0 & 0 & 0 & 0 & 0 & 0 & 0 & 0 & 0\\ 0 & 0 & 0 & 0 & 0 & 1 & 0 & 1 & 0 & 0 & 0 & 0 & 0 & 0 & 0 & 0 & 0 & 0 & 0\\ 0 & 0 & 0 & 0 & 0 & 0 & 0 & 0 & 1 & 0 & 0 & 1 & 0 & 0 & 0 & 0 & 0 & 0 & 0\\ 0 & 0 & 0 & 0 & 0 & 0 & 0 & 0 & 0 & 1 & 0 & 0 & 1 & 0 & 0 & 0 & 0 & 0 & 0\\ 0 & 0 & 0 & 0 & 0 & 0 & 0 & 0 & 0 & 0 & 0 & 0 & 0 & 1 & 0 & 1 & 0 & 0 & 0\\ 0 & 0 & 0 & 0 & 0 & 0 & 0 & 0 & 0 & 0 & 0 & 0 & 0 & 0 & 1 & 0 & 1 & 0 & 0\\ 0 & 0 & 0 & 0 & 0 & 0 & D_{1} & -D_{2} & D_{3} & -D_{4} & D_{5}-D_{6} & 0 & 0 & 0 & 0 & 0 & 0 & 0 & 0 \end{array}\right],$$

where $F_{\mathrm{ul}5}^{x}$ and $F_{\mathrm{ul}5}^{y}$ are the *x* and *y* components of the unlock actuator force when applied in the form $F_{\mathrm{ul}}\left(\begin{array}{c} F_{\mathrm{ul}5}^{x}\\ F_{\mathrm{ul}5}^{y} \end{array}\right)$, and

A 4 $$\begin{array}{rl} D_{1} & =\frac{L_{1}}{2}\sin\theta_{1}-l_{13}\sin(\theta_{1}+\psi_{13}),\\ D_{2} & =\frac{L_{1}}{2}\cos\theta_{1}-L_{13}\cos(\theta_{1}+\psi_{13}),\\ D_{3} & =\frac{L_{1}}{2}\sin\theta_{1}-L_{15}\sin(\theta_{1}+\psi_{15}),\\ D_{4} & =\frac{L_{1}}{2}\cos\theta_{1}-L_{15}\cos(\theta_{1}+\psi_{15}),\\ D_{5} & =F_{\mathrm{ul}1}^{x}\left(\frac{L_{1}}{2}\sin\theta_{1}-l_{\mathrm{ul}1}\sin(\theta_{1}+\psi_{\mathrm{ul}})\right)\\ \mathrm{and}\;D_{6} & =F_{\mathrm{ul}1}^{y}\left(\frac{L_{1}}{2}\cos\theta_{1}-l_{\mathrm{ul}1}\cos(\theta_{1}+\psi_{\mathrm{ul}})\right). \end{array}\}$$

Here, *F*_ul_ is the direction vector for the unlock actuator force, with the subscript number indicating the link the force acts on and the superscript indicating the coordinate direction in which it acts; *l*_ul1_ and *l*_ul5_ are the distances from the centres of gravity of link $L_{1}$ and link $L_{5}$ (respectively) to the point where the unlock actuator force acts on that link; and *ψ*_ul_ is the angle between *l*_ul1_ and link $L_{1}$'s centreline.
